# Clinical features and molecular characteristics of childhood community-associated methicillin-resistant *Staphylococcus aureus* infection in a medical center in northern Taiwan, 2012

**DOI:** 10.1186/s12879-017-2560-0

**Published:** 2017-07-05

**Authors:** Hong-Kai Wang, Chun-Yen Huang, Yhu-Chering Huang

**Affiliations:** 1 School of Medicine, Chang Gung University, Gueishan, Taoyuan, Taiwan; 2 Department of Pediatrics, Chang Gung Memorial Hospital at Linkou, No. 5, Fu-Shin Street, Gueishan, 333 Taoyuan, Taiwan

**Keywords:** Methicillin-resistant *Staphylococcus aureus*, Community-associated, Children, Taiwan, Sequence type 59

## Abstract

**Background:**

Since first reported in 2002, the rate of methicillin-resistant *Staphylococcus aureus* (MRSA) among childhood community-associated (CA) *S. aureus* infection in Taiwan increased significantly up to 2005. There have been no reports on this issue since then.

**Methods:**

We prospectively collected clinical *S. aureus* isolates from the patients <19 years of age in a university-affiliated hospital in 2012. Only first isolate from each patient was included. The medical records were retrospectively reviewed and the patients were classified as CA or healthcare-associated (HA) by the standard epidemiologic criteria. Isolates as CA-MRSA were further characterized by pulsed-field gel electrophoresis, staphylococcal cassette chromosome (SCCmec) typing, and multilocus sequence typing.

**Results:**

A total of 409 *S. aureus* isolates were included, and 260 (63.6%) were MRSA. The proportion of MRSA among all *S. aureus* isolates in 2012 increased significantly (*p* < 0.001) compared to that in 2004–2005. Of the 181 CA-MRSA isolates, 86.2% were identified from pus or wound. Nine pulsotypes were identified with two major types (type D, 119 (65.7%); type C, 27 (14.9%). Most of the isolates carried either SCC*mec* IV (66 isolates, 36%) or V_T_ (112 isolates, 62%). 128 isolates (71%) carried Panton-Valentine leukocidin (PVL) genes. Clonal complex (CC) 59 accounted for 146 isolates (80.7%) of two major pulsotypes, CC45 for 19 isolates, ST30 for 6 isolates and ST8 (USA 300) for 4 isolates. In addition to penicillin (100%), most isolates were resistant to erythromycin (81%) and clindamycin (79.3%).

**Conclusions:**

Around two-thirds of childhood community-associated *S. aureus* infections in northern Taiwan were MRSA. Though CC59 is still the prevalent community clone, several new clones emerged in northern Taiwan.

## Background

Methicillin-resistant *Staphylococcus aureus* (MRSA) is an important pathogen, which can cause various infectious diseases from mild skin and soft tissue infection (SSTI) to fulminant septicemia. Traditionally, MRSA is regarded as a hospital pathogen. Recent studies revealed an increasing rate of MRSA infection in individuals without risk factors predisposing for acquisition of MRSA, named as community-associated MRSA (CA-MRSA) [1–7]. Compared with healthcare-associated MRSA (HA-MRSA), CA-MRSA not only caused a different clinical disease entities but also had a different molecular characteristics [1–7]. Usually, the clinical manifestation of CA-MRSA infection is SSTI such as cellulitis or abscess [1–7]. HA-MRSA, in contrast, often causes pneumonia and sepsis. Genetically, CA-MRSA isolates are characterized by limited antibiotic resistance (except to β-lactams), and possess different exotoxin gene profiles (e.g. Panton-Valentine leukocidin genes, PVL).

In Taiwan, the predominant strain of CA-MRSA is sequence type (ST) 59/Staphylococcal chromosomal cassette (SCC) *mec* V_T_/ PVL-positive while HA-MRSA are ST239/SCC*mec* III/PVL-negative and ST 5/SCC*mec* II/PVL-negative in 2000s [5–9]. Since first report in 2002 [10], the rate of MRSA amongst childhood CA *S. aureus* infections increased significantly from 9.8% in 1999–2000 [10] to 55.7% in 2004–2005 [8]. However, there have been no reports published on this issue since 2005. Therefore, we conducted this study to re-evaluate if the epidemiology, clinical manifestations and molecular characteristics of childhood CA-MRSA infections changed in the past decade in Taiwan.

## Methods

This study was conducted in Chang Gung Memorial Hospital (CGMH) at Linkou, which is a university-affiliated teaching hospital in northern Taiwan and provides a range of care, from primary to tertiary care, with 3700 beds. Between 2006 and 2012, all the records of clinical *S. aureus* isolates, including MRSA, from children less than 19 years of age, excluding the isolates from neonatal units and survey for colonization, were extracted from the dataset of microbiology laboratory of CGMH. For the isolates identified in2008 and 2012, we retrospectively reviewed the medical records of the patients. If there were multiple episodes (isolates) collected from a single patient within the same calendar year, only the first episode (isolate) was included for analysis. We classified MRSA isolates into CA-MRSA and HA-MRSA according to the definitions proposed by Naimi et al. and HA infections were further categorized to hospital-onset (HO) and community-onset (CO). [2] Briefly, hospitalized patients infected by MRSA after 48 h of admission were classified as HO. For those identified within 48 h of admission, the patients with the risk factors including hospitalization, a permanent indwelling catheter, surgery, dialysis, and history of residence in a long-term-care facility within the previous 12 months, were categorized as CO-MRSA infection. In contrast, patients without above risk factors were regarded as community-associated (CA).

MRSA was identified according to Clinical and Laboratories Standards Institute (CLSI) guidelines [11]. Only MRSA isolates identified from CA infections in 2012 were included for further characterization. Antimicrobial susceptibility test was according to CLSI guidelines [11]. The molecular methods included pulsed-field gel electrophoresis (PFGE) with *Sma* I digestion, Staphylococcal cassette chromosome *mec* (SCC*mec*) typing, and detection of the Panton-Valentine leukocidin (PVL) genes. Some isolates of representative PFGE patterns were selected for further characterization by multilocus sequence typing (MLST), and spa typing. One locus difference in the MLST was categorized into the same clonal complex. All the molecular methods were described elsewhere previously [9, 12–17]. The results were analyzed by the chi-square test and statistically significance was defined as *p* < 0.05.

## Results

During the study period from 2006 to 2012, the yearly isolate numbers of *S. aureus* as well as MRSA from pediatric patients, excluding those hospitalized at neonatal units, are provided in Table 1. A total of 2078 *S. aureus* isolates were identified and overall, the proportion of MRSA was 63%. The yearly *S. aureus* isolates ranged from 140 isolates in 2011 to 409 isolates in 2012 and the proportion of MRSA was from 61.2% in 2009 to 69.3% in 2007 and 2011, respectively. In 2008, 142 (53.6%) of 265 *S. aurues* isolates were CA and 89 (62.7%) of 142 CA *S. aureus* isolates were MRSA; while in 2012, 280 (68.5%) of 409 *S. aurues* isolates were CA and 181 (64.6%) of 280 CA *S. aureus* isolates were MRSA.

**Table 1:** Distribution of clinical *Staphylococcus aureus* and methicillin-resistant *S. aureus* (MRSA) isolates from pediatric patients in Chang Gung Memorial hospital from 2004 to 2012

Year	All isolates, No. (%)		Community-associated, No. (%)	
	*S. aurues*	MRSA	*S. aurues*	MRSA
2004.7–2005.6 [8]	357	173 (48.5)	183	102 (55.7)
2006	246	167 (67.9)		
2007	313	217 (69.3)		
2008	265	172 (64.9)	142	89 (62.7)
2009	165	101 (61.2)		
2010	183	122 (66.7)		
2011	140	97 (69.3)		
2012	409	260 (63.6)^#^	280	181 (64.6)*
Total	2078	1309 (63.0)		

^*^the rate significantly increased from 2004 to 2005 to 2012, *p* = 0.034

^#^the rate significantly increased from 2004 to 2005 to 2012, *p* < 0.001

Among the 260 MRSA isolates identified in 2012, 181 isolates (70%) were recognized as community-associated. Table 2 illustrates the distribution of MRSA stratified by the sources of specimens. CA-MRSA isolates were mainly collected from pus (86.2%) whereas HA-MRSA isolates were mostly collected from pus (48.1%) and sputum (25.3%). Among 181 patients infected by CA-MRSA, 157 (86.8%) presented with skin and soft tissue infection, 13 (7.2%) presented with urinary tract infection, 6 (3.3%) presented with pneumonia, 3 (1.7%) presented with bacteremia, and one (0.6%) each with conjunctivitis, and keratitis, respectively.

**Table 2:** Distribution of 260 clinical methicillin-resistant *Staphylococcus aureus* stratified by origin of specimens

Origin	Community-associated No. (%)	Healthcare-associated No. (%)	*p*-value	Healthcare-associated, No. (%)		
				Community-onset	Hospital-onset	*p*-value
No. of isolates	181	79	-	50	29	-
Blood	3 (1.7)	4 (5.1)	0.128	1(2)	3(10.3)	0.137
Sputum	6 (3.3)	20 (25.3)	<0.001	8(16)	12(41.4)	0.014
Pus	156 (86.2)	38(48.1)	<0.001	31(62)	7(24.1)	0.001
CVC	0	1(1.3)	0.304	0(0)	1(3.4)	0.367
Urine	13 (7.2)	6 (7.6)	0.544	5(10)	1(3.4)	0.278
DTS	0	0	1	0	0	1
Ascites	0	3(3.8)	0.027	3(6)	0(0)	0.248
CSF	0	0	1	0	0	1
Others	3^a^ (1.7)	7^b^ (8.9)	0.01	2(4)	5 (17.2)	0.059

*CVC* central venous catheter, *DTS*, deep tissue specimen, *CSF* cerebrospinal fluid

^a^conjunctiva, 1; eye secretion, 1; other, 1

^b^bronchoalveolar lavage, 1; central venous catheter tip, 4; others, 2

The antimicrobial susceptibility results of the 181 CA-MRSA isolates are shown in Table 3. All of the CA-MRSA isolates were susceptible to vancomycin, teicoplanin, doxycycline but resistant to penicillin. There was only one strain resistant to trimethoprim–sulfamethoxazole and fusidic acid. Most isolates were resistant to erythromycin (81%) and clindamycin (79.3%).

**Table 3:** Comparison of antibiotic resistance between 181 community-associated methicillin-resistant *Staphylococcus aureus* isolates from 2012 and 102 CA-MRSA isolates from 2004 to 2005

Year	Total	Penicillin	Erythromycin (*n* = 169) ^*^	Clindamycin (*n* = 169) ^**^	Fusidic acid	Trimethoprim /sulfamethoxazole***	Tigecycline	Linezolid	Vancomycin	Teicoplanin
2012	181	181(100)	137(81.1)	134(79.3)	1(0.6)	1(0.6)	0(0)	0(0)	0(0)	0(0)
2004–2005 [8]	102	102(100)	96(94.1)	94(92.2)	-	5(4.9)	-	-	0(0)	0(0)

All the isolates were resistant to penicillin while susceptible to tigecycline, linezolid, vancomycin and teicoplanin

*p = 0.003

**p = 0.01

***p = 0.024

The molecular characteristics of 181 CA-MRSA isolates are illustrated in Table 4. There were nine pulsotypes with two major types (type D, 119 (65.7%); type C, 27 (14.9%)). Three SCC*mec* types were identified and SCC*mec* type V_T_ accounting for 61.9% of the isolates outnumbered the others. SCC*mec* type IV accounted for 36.5% of the isolates.70.7% of the CA-MRSA isolates carried PVL genes. All but one isolates of pulsotype D carried PVL genes. For MLST, there were totally 6 sequence types identified. Clonal complex 59(CC59) and clonal complex 45(CC45) were the main MLST types(complexes) and accounted for 80.7% and 10.5% of the isolates, respectively. 29 CA-MRSA isolates were selected for Spa typing and 12 Spa types were identified. Table 5 shows the major clones of CA-MRSA and they are ST59/Pulsotype D/SCC*mec* V_T_/PVL-positive, ST59/Pulsotype C/SCC*mec* IV/ PVL-negative and ST45/Pulsotype AK/SCC*mec* IV/PVL-negative.

**Table 4:** Molecular characteristics of 181 community-associated methicillin-resistant *Staphylococcus aureus* isolates stratified by the pulsed-field gel electrophoresis patterns

Characteristics	Pulsed-field gel electrophoresis patterns								
	C	D	U	AK	AG	AI	AF	AQ	BM
No. isolates	27 (14.9)	119 (65.7)	3 (1.7)	17 (9.4)	6 (3.3)	4 (2.2)	2 (1.1)	1 (0.6)	2 (1.1)
SCC*mec* type	IV	V_T_ (112), IV (6), V (1)	IV	IV	IV	IV	II, IV	IV	IV, V
PVL-positive	0	118	0	0	6	4	0	0	0
Sequence type	59 (4/5), 2844^a^ (1/5)	59 (4/5), 2842^a^ (1/5)	573 (1/1)	45 (1/3), 508^b^ (2/3)	30 (2/2)	8 (1/1)	89 (1/1)	2843^c^	45 (1/1)
Spa type	t437 (5/6), t441^d^ (1/6)	t437 (7/9), t441 (1/9), t4145^d^ (1/9)	t3525 (1/1)	t026 (1/4), t015 (2/4), t550 (1/4)	t019 (2/3), t1752^e^ (1/3)	t008 (2/2)	t375 (1/1)	t015 (1/1)	t1081 (2/2)

SCC*mec*, staphylococcal chromosomal cassette; PVL, Panton-Valentine leukocidin; UT, untypeable

^a^Single locus variant of ST59; ^b^single locus variant of 45; ^c^single locus variant of 508; ^d^clonal complex of t437; ^e^clonal complex of t019

**Table 5:** Comparison of major clones from community-associated methicillin-resistant *Staphylococcus aureus* isolates

Year	No. of isolates	ST59/PFGE D/ SCC*mec* V_T_ /PVL+	ST59/PFGE C/ SCC*mec* IV/ PVL-	ST45/PFGE AK/ SCC*mec* IV/ PVL-	ST30/PFGE AG/ SCC*mec* IV/ PVL+	ST 239/PFGE A/ SCC*mec* III/ PVL-
2012	181	111 (61.3)	27 (14.9)	17 (9.4)	6 (3.3)	0
2004.07–2005.06 [8]	173	70(69)	9(8.8)	0	0	4(3.9)
Spa typing		t437 (7/9), t441 (1/9), t4145^d^ (1/9)	t437 (5/6), t441^d^ (1/6)	t026 (1/4), t015 (2/4), t550 (1/4)	t019 (2/3), t1752^e^ (1/3)	

Values are given as n (%)

*PFGE* pulsed-field gel electrophoresis, SCC*mec* staphylococcal chromosomal cassette, *PVL* Panton–Valentine leukocidin, *MLST* multilocus sequence typing

^a^A significant difference was found between the community-associated and healthcare-associated isolates in respect to PFGE D, PFGE A, and PFGE F clones (*p* < 0.001)

^b^A significant difference was found between the community-onset and hospital-onset isolates in terms of the third clone (*p* = 0.022)

## Discussion

Comparing the results from a previous study [8], which was conducted in a similar design and definition in the same hospital during 2004–2005, we found that the proportion of MRSA among the clinical *S. aureus* isolates from children in our hospital increased from 48.5% in 2004–2005 to >60% between 2006 and 2012 with the highest (69%) in 2007 and 2011, respectively (Table 1). Likewise, the proportion of MRSA among community-associated *S. aureus* infections in children increased from 55.7% in 2004–2005 [8] to 62.7% in 2008 and 64.6% in 2012 (Table 1). Though still going up since 2005, the rate seemed to reach a plateau up to 2012. These findings correlated with the rising trend of nasal MRSA colonization among pediatric population in northern Taiwan [5–7].

The rate of MRSA among community-acquired *S. aureus* infection varies markedly worldwide, ranges from <1% to >50% in different countries and is higher in children than in adults [3–7]. In some countries such as the United States, Taiwan, Canada, and Australia, CA-MRSA infections in patients is common, while in Europe it is low but increasing. A prospective surveillance study conducted, 2004 ~ 2006, in eight Asian countries [18] reported that MRSA accounted for 25.5% of 1463 isolates of CA *S. aureus* infections, and a rate > 30% was noted in Taiwan, the Philippines, Vietnam and Sri Lanka.

Consistent with those reported previously [1–9], most CA-MRSA isolates were identified from pus (86.2%) while the source for HA-MRSA isolates were relatively broader, including sputum (25.3%), pus (48.1%), urine (7.6%), blood 5.1%) and CVP tips (5.1%). Furthermore, among HA-MRSA isolates, CO-MRSA isolates were more likely to be identified from pus (62%) while HO-MRSA isolates were more likely to be identified from sputum (41.4%) and only 24.1% from pus. This origin profiles again indicated that CA-MRSA infections usually presented with skin and soft tissue infections whereas HA-MRSA infections often presented with a diverse spectrum of disease entities.

In this study, the clone characterized asST59/SCC*mec* V_T_/PVL-positive, named as Taiwan clone, was the predominant clone of CA-MRSA isolates, which is consistent with previous study [8]. The dominance of this clone among CA-MRSA isolates in Taiwan persisted for more than one decade since identified [5–9, 19–21]. In contrast, sequence type ST239/SCC*mec* III/ PVL-negative, which was the endemic HA clone and accounted for nearly 5% of all CA-MRSA isolates in the previous study [8], was not identified in this study (Table 5). However, several clones which were rarely reported in Taiwan previously were identified in this study and included ST8/t008/SCC*mec* IV/PVL-positive (USA 300), clonal complex 45/ SCC*mec* IV/ PVL-negative and ST 30/t019/SCC*mec* IV/ PVL-positive. ST 30 is the major CA-MRSA clone prevailing in southeastern Asian countries, Australia and Japan [6, 7]. ST8 (USA 300) is the major CA-MRSA clone prevailing in Canada and USA and has been reported from some Asian countries recently [6, 7]. Reviewing the medical records of the patients infected with both clones, we did not identify any obvious travel history or contact history. The issues regarding how did these clones appear and evolve in Taiwan and what is the impact of these clones in Taiwan need further observations. Also, continuing surveillance is needed.

In this study, around 80% of CA-MRSA isolates were resistant to erythromycin and clindamycin. The resistant rates to both antibiotics, though still high, seemed to decrease gradually, compared with those in 2004–2005 (Table 3). Likewise, all the isolates were still susceptible to vancomycin, teicoplanin, linezolid and doxycycline and all but one isolates were susceptible to trimethoprim/sulfamethoxazole and fusidic acid. The antibiotic resistant pattern is correlated with the molecular pattern of the CA-MRSA isolates in different years [5–9].

There are several limitations in this study. First, this study was conducted in a single medical center and the epidemiologic features shown here may not represent the whole perspective in Taiwan. However, our hospital is the largest hospital in Taiwan and the case number in this study was not small, so it still can partly reflect the current status of childhood CA-MRSA in Taiwan. Second, though the isolates were prospectively collected, medical records of the patients were retrospectively reviewed, so some risk factors for MRSA acquisition in the patients may be missed and thus the patients in HA-MRSA group might be misclassified to CA-MRSA group. However, no genetically HA-MRSA isolate was identified from the patients in CA-MRSA group. Third, some of the specimen types e.g. pus, wound swabs and sputum may be easily contaminated by *Staphylococcus aureus*. Thus, whether or not all *Staphylococcus aureus* isolates identified in this study caused active infection is somewhat questionable. However, all the isolates were clinical isolates from the patients with active diseases and were treated as such. Last, only one-year CA-MRSA isolates identified in 2012 underwent molecular characterizations, so the trend of molecular epidemiology of CA-MRSA as well as HA-MRSA in northern Taiwan could not be clearly shown here. Further studies are needed.

## Conclusion

In conclusions, the proportion of MRSA among childhood community-associated *S. aureus* infections in northern Taiwan increased significantly from <10% in 1999–2000 to more than half in 2004–2005 and then reached the plateau (>60%)up to 2012. Clonal complex 59 has been the predominant community clone since identified in Taiwan; however, several other community clones such as ST30 and USA 300 emerged. Continued surveillance is needed.

## Abbreviations

CA: Community-associated
CC: Clonal complex
CLSI: Clinical and Laboratories Standards Institute
CO: Community-onset
HA: Healthcare-associated
HO: Hospital-onset
MLST: Multilocus sequence type
MRSA: Methicillin-resistant *Staphylococcus aureus*
MSSA: Methicillin-sensitive *Staphylococcus aureus*
PFGE: Pulsed-field gel electrophoresis
PVL: Panton-Valentine leucocidin
SCC: Staphylococcal chromosomal cassette
